# Adhesive Capsulitis in a Postmenopausal Woman With Thyroid Dysfunction

**DOI:** 10.7759/cureus.102598

**Published:** 2026-01-29

**Authors:** Tanner F Wise, Joe Bhagratie, Katherine Lemus, Fiona Lin, Stephen Westfall

**Affiliations:** 1 Medicine, Lake Erie College of Osteopathic Medicine, Bradenton, USA; 2 Orthopedics, Baptist Medical Center Jacksonville, Jacksonville, USA; 3 Family Medicine, Ascension St. Vincent’s Family Medicine Center, Jacksonville, USA; 4 Internal Medicine, Lake Erie College of Osteopathic Medicine, Bradenton, USA

**Keywords:** adhesive capsulitis, estrogen deficiency, frozen shoulder, hydrodistention, menopause, suprascapular nerve block

## Abstract

Adhesive capsulitis, also known as frozen shoulder, is characterized by progressive pain and loss of shoulder mobility due to capsular inflammation and fibrosis. This is a case of a 50‑year‑old postmenopausal woman with a history of Graves’ disease treated by radio‑iodine ablation who presented with a year‑long history of gradually worsening left‑shoulder pain and stiffness. Imaging revealed mild acromioclavicular arthrosis and supraspinatus tendinosis without rotator cuff tear, findings consistent with adhesive capsulitis. Conservative management with physiotherapy, home exercise, and non‑steroidal anti‑inflammatory drugs provided little relief, and a subacromial corticosteroid injection gave temporary improvement. The patient subsequently underwent ultrasound‑guided glenohumeral hydrodistention combined with a suprascapular nerve block and intra‑articular corticosteroid, allowing for immediate pain relief and increased passive range of motion, enabling more effective participation in physical therapy. This case shows how thyroid imbalance and reduced estrogen may jointly contribute to capsular fibrosis and demonstrates that hydrodistention with a nerve block can be used as an effective treatment for adhesive capsulitis refractory to conservative management. Early identification of endocrine and hormonal risk factors may facilitate timely intervention and improve outcomes for patients with frozen shoulder.

## Introduction

Adhesive capsulitis, or frozen shoulder, is a condition of the glenohumeral joint marked by progressive pain and a significant loss of both active and passive range of motion, with stiffness that is notably out of proportion to the level of pain. The underlying pathology involves capsular inflammation, synovial proliferation, and subsequent fibrosis of the capsule, which ultimately leads to joint contracture. If left untreated, adhesive capsulitis can result in deltoid and rotator cuff muscle atrophy due to prolonged lack of mobility and disuse, especially in patients with other comorbidities and advanced systemic diseases [1,2]. While many cases are idiopathic, emerging evidence implicates systemic endocrine and hormonal conditions, particularly thyroid disease and menopause, in the etiology and chronicity of adhesive capsulitis.

Thyroid hormone imbalance can disrupt collagen turnover and extracellular matrix homeostasis, promoting fibroblast proliferation and connective tissue thickening [3,4]. For example, hypothyroidism and subclinical hypothyroidism nearly double the risk of adhesive capsulitis compared with normal thyroid function [3,5]. This association is thought to arise from systemic inflammation and metabolic dysfunction, as shown in increased inflammatory markers and dyslipidemia. Reduced thyroid hormone activity disrupts immune regulation and fibroblast function, creating a pro-inflammatory environment that promotes abnormal collagen deposition and subsequent capsular fibrosis.

Likewise, estrogen deficiency plays a role in the pathogenesis of adhesive capsulitis; after menopause, estrogen deficiency reduces the hormone’s anti‑inflammatory and anti‑fibrotic properties, increasing expression of profibrotic cytokines such as interleukin (IL)-6 and transforming growth factor-beta (TGF‑β) [4,6]. In a premenopausal patient, estrogen, specifically estradiol, inhibits fibroblast activation and extracellular matrix deposition in the shoulder capsule via the G protein-coupled estrogen receptor (GPER) and suppression of the phosphoinositide 3-kinase-Akt (PI3K-Akt) signaling pathway. After menopause, estrogen deficiency counteracts this inhibitory effect, resulting in elevated fibroblast activity, collagen production, and ultimately, capsular fibrosis characteristic of adhesive capsulitis [7].

## Case presentation

A 50‑year‑old woman presented with a one‑year history of gradually worsening pain and stiffness in her left shoulder. Symptoms began insidiously while performing morning yoga and progressed over six months to significant pain with overhead activity, an inability to perform push‑ups, and difficulty reaching behind her back. She denied trauma or prior shoulder pathology. The pain radiated into the neck and arm without neurologic deficits.

Past medical history and medications

The patient’s history was notable for Graves’ disease treated with radio‑iodine ablation; she was on levothyroxine and liothyronine replacement therapy. Other conditions included osteopenia, irritable bowel syndrome, sicca syndrome, vitamin D deficiency, and chronic anxiety. She was menopausal and managed with a combination of estradiol, progesterone, and testosterone replacement therapy. Medications comprised levothyroxine (125-137 μg daily), liothyronine (5 μg daily), progesterone (100 mg daily), transdermal estradiol (0.1 mg/24 hr), escitalopram (20 mg daily), alprazolam (0.5 mg as needed), and high‑dose vitamin D₂ (50000 IU weekly).

Imaging and initial management

**Figure 1:**
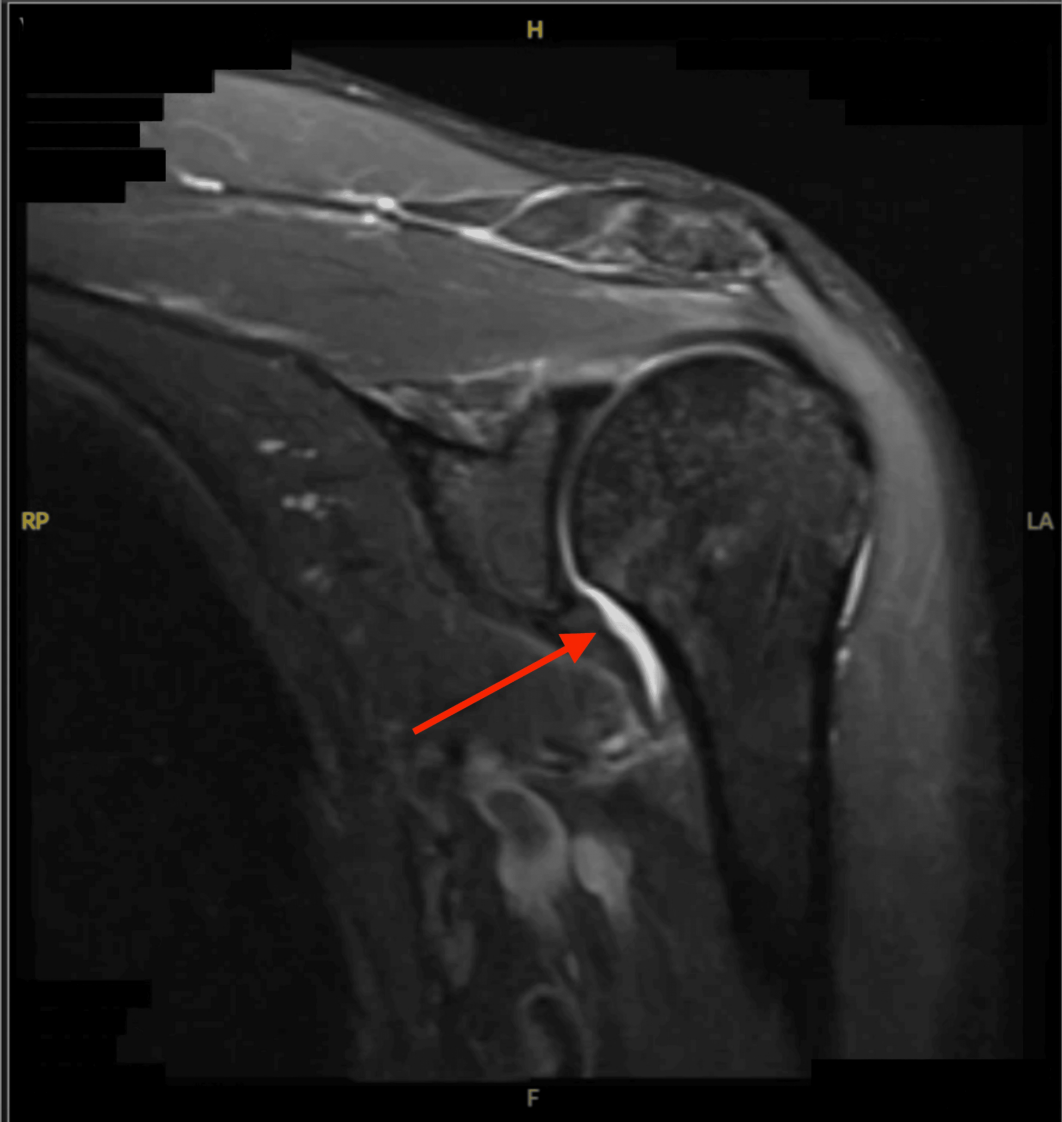
Sagittal T2-weighted MRI of the left shoulder demonstrating supraspinatus tendinosis with fluid signal in the subacromial space and capsular changes consistent with adhesive capsulitis MRI: magnetic resonance imaging; H: head; F: feet; LA: left anterior; RP: right posterior

Magnetic resonance imaging (MRI) of the left shoulder performed in August 2025 showed mild acromioclavicular joint arthrosis, a type II acromion with mild supraspinatus outlet narrowing according to the Bigliani classification, and insertional tendinosis of the supraspinatus without evidence of partial- or full‑thickness rotator cuff tear [8]. These findings were consistent with adhesive capsulitis (Figure 1). All clinical assessments were based on standard physical examination findings and imaging interpretation. The patient underwent supervised physiotherapy twice weekly with home exercises for several months; non‑steroidal anti‑inflammatory drugs (NSAIDs) provided limited relief. A landmark‑guided subacromial corticosteroid injection (40 mg triamcinolone with ropivacaine) produced only transient improvement.

Definitive intervention

Due to persistent pain and restricted mobility, the patient underwent ultrasound‑guided glenohumeral hydrodistention combined with a suprascapular nerve block on October 21, 2025. Under sterile conditions, a suprascapular nerve block was performed using 1% lidocaine. The hydrodistention injectate consisted of 5 mL of 1% lidocaine, 40 mg of triamcinolone acetonide, 1 mL of 0.2% ropivacaine, and 17 mL of 10% dextrose. The fluid was injected via a posterior approach under ultrasound guidance, with gentle capsular distention and manipulation. The patient reported immediate pain relief and improved passive range of motion, and began physical therapy the same day. At a two‑week follow‑up, she reported modest gains in range of motion and improved tolerance to therapy, and a follow-up visit was scheduled for six to eight weeks.

## Discussion

Thyroid dysfunction and adhesive capsulitis

Both hypothyroid and hyperthyroid conditions have been associated with an increased prevalence of adhesive capsulitis. Alterations in thyroid hormone levels can disrupt collagen synthesis and glycosaminoglycan turnover, leading to thickening of periarticular connective tissues [3,5]. Autoimmune thyroid disorders, such as Graves’ disease, amplify systemic inflammation and may activate fibroblasts, contributing to capsular fibrosis. Thyroid hormones, especially in excess, modulate immune cell proliferation, cytokine release (including interferon-gamma (IFN-γ), tumor necrosis factor-alpha (TNF-α), IL-6, IL-8, and IL-17A), and antibody production, such as thyrotropin receptor antibody (TRAb), which can promote a pro-inflammatory state [9,10]. Meta‑analyses have shown a statistically significant association between thyroid disease and adhesive capsulitis, with hypothyroid patients demonstrating slower recovery and worse shoulder function [3,11]. While prior literature predominantly emphasizes the association between adhesive capsulitis and hypothyroidism, comparatively little evidence addresses the potential contribution of hyperthyroid states. This case draws attention to hyperthyroid disease as a potential contributor to adhesive capsulitis.

Menopause and estrogen deficiency

The incidence of adhesive capsulitis peaks in women between 40 and 60 years of age. Estrogen plays an anti‑inflammatory and anti‑fibrotic role within connective tissues by modulating cytokines and reducing fibroblast proliferation [4,6]. Following menopause, estrogen decline or receptor desensitization increases the expression of profibrotic cytokines such as TGF‑β and IL‑6, promoting synovial and capsular thickening. Studies report a higher prevalence of frozen shoulder in perimenopausal and postmenopausal women; even with hormone replacement therapy, local receptor regulation may not fully restore protective effects [4,6]. In this case, the patient’s combined endocrine (thyroid) and hormonal (menopausal) factors likely created a synergistic effect that predisposed her to adhesive capsulitis and hindered recovery.

Hydrodistention with suprascapular nerve block

Hydrodistention involves injecting a relatively large volume of fluid into the glenohumeral joint under imaging guidance to distend the capsule, mechanically disrupt adhesions, and flush inflammatory mediators. Multiple studies demonstrate that hydrodistention, particularly when combined with intra‑articular corticosteroid and followed by early mobilization, provides significant improvements in pain and range of motion compared with corticosteroid injection alone [12]. The suprascapular nerve block reduces procedural discomfort and allows patients to tolerate immediate physiotherapy, which is essential for maintaining gains in motion. In this case, hydrodistention led to rapid pain relief and early improvements in motion, facilitating rehabilitation.

Clinical implications

This case underscores the connection between endocrine and hormonal influences with musculoskeletal disorders. In patients presenting with adhesive capsulitis, clinicians should inquire about thyroid status and menopausal symptoms and ensure optimization of thyroid replacement therapy and assessment of hormonal management. Early identification of systemic risk factors may prompt more aggressive interventions, such as hydrodistention with nerve block, and targeted patient counselling, potentially improving outcomes and reducing chronic disability.

## Conclusions

Adhesive capsulitis is a multifactorial condition in which local capsular inflammation and fibrosis can be exacerbated by systemic endocrine and hormonal dysregulation. In this postmenopausal woman with a history of Graves’ disease, the interplay between thyroid dysfunction and estrogen deficiency likely contributed to the development and persistence of her frozen shoulder, which initially manifested as significant pain with overhead activity, inability to perform push-ups, and difficulty reaching behind her back. Ultrasonography-guided hydrodistention combined with a suprascapular nerve block provided rapid pain relief and early improvement in range of motion, enabling more effective participation in physical therapy. Recognizing systemic contributors and implementing comprehensive management strategies, including optimization of thyroid function, evaluation of hormone therapy, and timely interventional procedures, may improve outcomes for patients with adhesive capsulitis.
